# Effects of ZSM-5 zeolite on pyrolysis of polystyrene: from stabilizing to catalyzing

**DOI:** 10.55730/1300-0527.3574

**Published:** 2023-06-07

**Authors:** Qing-yuan MA, Zhen HUANG, Xuan REN, Jia-jia ZHAO, Fu CHEN, Li-jun TENG

**Affiliations:** Department of Packaging Engineering, Tianjin University of Commerce, China

**Keywords:** Polystyrene, ZSM-5 zeolite, pyrolysis, thermogravimetric analysis, kinetic parameters, PS/ZSM-5 hybrids

## Abstract

Nonisothermal pyrolysis measurements of polystyrene (PS)/ZSM-5 zeolite hybrids are conducted in N_2_ and thermogravimetric results have been kinetically analyzed with different isoconversional methods. Experimental results show that the addition of 5 and 10 wt.% ZSM-5 zeolite has increased the initial pyrolysis temperature of PS while the addition of 20 and 30 wt.% ZSM-5 zeolite can significantly decrease the initial pyrolysis temperature of PS. Elevated activation energy is resulted by adding low zeolite amount whereas reduced activation energy is obtained by adding high ZSM-5 amounts. The effect of zeolite ZSM-5 on PS pyrolysis can thus be observed to transfer from stabilizing to catalyzing. Furthermore, the pyrolysis mechanism functions of PS/zeolite hybrids are determined by integrating the master plots method with a new compensation effect method, and the most appropriate reaction models are found to be F0.92, F0.85, F0.56 and A1.32 for describing the pyrolysis of the PS/ZSM-5 hybrids with a zeolite loading of 5, 10, 20 and 30 wt.%, respectively. With the kinetic parameters thus available, the temperature-dependent mass conversion curves have been recast, leading to satisfactory simulations for PS/ZSM-5 hybrids.

## 1. Introduction

Plastic packaging products have been used extremely widely in the world because of excellent performances such as lightness in weight, good wear resistance, mechanical properties and low cost. However, these petroleum-based plastic packaging wastes are difficult to decompose naturally in the surrounding environment, resulting in the accumulation of a large amount of plastic wastes. Therefore, solving the waste problem due to the roaring-up of plastic packaging wastes has attracted people’s attention all over the world including China [1]. Among various plastic wastes, polystyrene (PS) is one of them since it is daily produced from heavily used cushioning packaging materials and milk-bottle materials [2]. However, there are still technical difficulties in the recycling of PS wastes, and now pyrolysis treatment is a very common technology for the treatment of plastic packaging wastes [3], and gaseous and liquid products from pyrolysis of PS wastes are fuel and chemical raw materials [4,5], so as to achieve the purpose of environmental protection and sustainable clean production. Therefore, an in-depth study on the pyrolysis process of PS will be helpful to better understand the significance for the application of pyrolysis technology in the treatment of PS packaging waste.

Thermogravimetric (TG) analysis and its first derivative (DTG) have been widely used in the analysis of pyrolysis degradation characteristics and decomposition mechanism of plastic wastes. The analysis of pyrolysis kinetics is mainly reflected in the accurate calculation of three kinetic factors, namely the apparent activation energy (*E_k_*), reaction model (*f*(*α*)) and preexponential factor (*A*). The International Confederation for Thermal Analysis and Calorimetry (ICTAC) Kinetics Committee [6] strongly has recommended that isoconversional methods be used to accurately calculate three kinetic parameters. Up to now, a number of scientists have conducted pyrolysis studies of polystyrene [7,8] and its wastes [9,10] and these studies show that high temperature pyrolysis of PS has produced styrene and other aromatic substances of great economic value for industrial applications. Furthermore, Nisar et al. [11] have found that the pyrolysis products of PS wastes have similar composition to those of diesel oil, gasoline, and kerosene, with many hydrocarbons from C2 to C15 in the liquid phase and mainly methane and ethane in the gas phase. Apart from the analysis of pyrolysis production, kinetic pyrolysis studies of PS plastics have also been conducted by many researchers [11, 12, 13, 14]. For examples, Ali et al. [12] have kinetically analyzed pyrolysis of PS waste and obtained activation energies of 99.4–149.2 kJ/mol and preexponential factor values of 2.9 × 10^7^–2.3 × 10^11^ while in their sister paper [11] *E*_k_ and *A* are found in the range of 82.3–202.8 kJ/mol and 3.5 × 10^6^–7.6 × 10^14^ min^−1^, respectively. Zhang et al. [13] have extensively conducted pyrolysis analysis of PS waste with a number of kinetic methods, resulting in the *E*_k_ of 125–147 kJ/mol, the ln*A* of 17–21 s^−1^, a reaction mechanism function of *f*(*α*) = (1 − *α*)^3/4^ and a differential rebuilding function of d*α/*d*t* = 2.18 *×* 10^8^exp(− 1.38 *×* 10^5^/R*T*)*α*^0.0309^(1 − *α*)^0.7689^. Ren et al. [14] have compared pyrolysis of expanded polystyrene (EPS) waste and Yakult milk bottle polystyrene (YPS) along with pure PS and found that EPS is easier to undergo pyrolysis than pure PS while YPS is more stable than pure PS. The activation energies by Coats-Redfern method are 127.9, 139.1, and 181.7 kJ/mol for EPS, pure PS, and YPS, respectively. In addition, the reaction mechanism function of pure PS, EPS, and YPS is determined to be respectively F0.58, F1.14, and F0.61 by using the combined Z-master plots method with differential composite method.

In the meantime, many attempts have been continuously made to study the effects of zeolite and metal oxide additives on thermal pyrolysis characteristics and kinetic processes of polymers [15, 16, 17, 18, 19, 20]. López et al. [15] investigated the effects of ZSM-5 zeolite and red mud catalysts on the pyrolysis process of plastic waste, and the results showed that the pyrolysis products were strongly dependent on ZSM-5 zeolite and the yield of pyrolysis liquid products has been enhanced with the use of zeolite. In the work of Wang et al. [16], nanosized ZnO and TiO_2_ are both seen to catalytically affect pyrolysis of poly lactic acid (PLA), as reflected by the reduced pyrolysis temperatures and decomposition activation energies. The authors have reported that the pyrolysis *E*_k_ of PLA is reduced by 11–32 kJ/mol for adding TiO_2_ and 35–59 kJ/mol for adding ZnO [16]. More recently, Patnaik et al. [17] have studied the effects of zeolite A on the pyrolysis of PS and other plastic wastes and found that zeolite A has promoted the pyrolysis of all plastic waste, and the activation energy of PS has significantly reduced from 295.0 to 230.6 kJ/mol, and the pyrolysis reaction order has changed from 1.5 to 1. Similarly, catalytic effect of zeolite A has also been reported on pyrolysis of polysulfone [18]. These results suggested that by means of catalytic impact, the energy consumption of pyrolysis process may be reduced, and the process may consequently become economical viable.

On the contrary, Farha et al. [19] have investigated the effect of nanosized ZnO on the pyrolysis of polystyrene but the results showed that the thermal stability of PS nanocomposites was higher than that without ZnO. Likewise, ZSM-5 zeolite has been reported to substantially improve the thermal stability of PLA and the pyrolysis *E*_k_ has increased from 99.7 to 159.48 or 153.79 kJ/mol after 10 and 20 wt.% ZSM-5 zeolite [20]. Therefore, it is of importance to properly choose a catalytic additive for pyrolysis of given polymers, otherwise, the opposite consequence will be obtained at the cost of large energy consumption.

These studies, even though, have demonstrated that the addition of an additive such as zeolite has a vital impact on the pyrolysis characteristics and kinetics of various plastic wastes including PS, the following concerns about the pyrolysis of PS still need to be investigated: (1) catalytic influences of the additives on the pyrolysis features and degradation mechanism of PS; (2) probable kinetic degradation model for the pyrolysis of PS; (3) calculation of kinetic parameters based on multiple heating rates of < 20 K/min; (4) verification and usability of three kinetic parameters. At present work, ZSM-5 (Zeolite Socony Mobil–5), a porous aluminosilicate belonging to the MFI family of zeolite [21], has been considered to be an additive to pyrolysis of PS. Earlier, Miskolczi et al. [22] have fully studied catalytic pyrolysis of PS by coarsely adding 2 wt.% of ZSM-5 zeolite. Apart from the yields and compositions of pyrolysis products, they have also performed rather simple kinetic analysis by assuming the first-order reaction model and found that when using ZSM-5 the *E*_k_ value of PS can be decreased by around 40 kJ/mol. However, the effect of higher ZSM-5 content on pyrolysis of PS has not been further reported yet, and therefore, the objective of present study is to investigate the effects of high ZSM-5 zeolite contents of 5–30 wt.% on pyrolysis of PS in nitrogen atmosphere. Then the pyrolysis features and kinetic parameters of PS and its ZSM-5 hybrids have been evaluated and compared for understanding the effect of the added ZSM-5 zeolite. Kinetically, the *E*_k_ has isoconversionally calculated by using model-free methodologies, which include integral methods like Flynn-Wall-Ozawa (FWO) [23,24], Coats-Redfern (CR) [25], Madhusudanan-Krishnan-Ninan (MKN) [26], Starink (SK) [27], Tang et al. (TLZW) [28] and Vyazovkin-Dollimore (VD) [29] methods, and differential Friedman (FD) method [30]. Furthermore, the most probable *f*(*α*) and ln*A* have been resulted by combining the master plots method [31] and a new way to obtain the compensation effect relationship [32]. Finally, a reconstruction of *α–T* curves for comparing against experimental results has been made for providing detailed information about kinetic pyrolysis behaviors of PS necessary for designing any industrial applications.

## 2. Experimental

### 2.1 Materials

Original polystyrene (PS) with a number average molecular weight of 100,000 was provided from Sinopec Guangzhou Branch, China and ZSM-5 zeolite of a Si/Al atomic ratio of 25 and a particle size of about 1 μm was purchased from Tianjin Nankai University Catalyst Factory, China. Trichloromethane (CHCl_3_) of 99% purity was purchased from Tianjin Kaitong Chemical Reagent Co. Ltd., China. ZSM-5 zeolite powder before usage was dehydrated at 623 K for 2 h and it was quickly kept in a dryer for preservation after naturally cooling down to ambient.

### 2.2 Sample preparation

At present work, PS/ZSM-5 hybrids were prepared as follows: At first, a certain amount of zeolite powder was weighed and added into 50 mL CHCl_3_ in a round-bottom flask under magnetic stirring for 30 min. Later on, about 1/3 amount of weighed PS was added and after continuous stirring for 1 h, the left PS of around 10 g was added to the resultant suspension. Another 2 h stirring was used to render zeolite particles evenly dispersed in the suspension. Then the suspension was poured onto the clean glass plate in a fume hood and flatted with a scraper for solvent evaporation. After the solvent was mostly evaporated, the film-like sample was peeled off from the glass plate and heated in a drying oven at 393 K for 1 h to completely remove any solvent residual. Finally, the sample was taken out of the oven and quickly kept in a desiccator for subsequent measurements. The hybrid samples loaded with 5, 10, 20 and 30 wt.% ZSM-5 zeolite were named Z-5, Z-10, Z-20, and Z-30, respectively.

The aboveprepared PS/ZSM-5 hybrids along with ZSM-5 powder and pure PS were subjected via X-ray diffraction (XRD) analysis to a Japan Rigaku D/max 2500v/PC X-ray diffractometer to characterize microscopic structures of all samples, and the diffraction angle was scanned in the range of 2θ = 5–50° at a speed of 4°/min. The XRD results can be used to study whether the crystalline structure of PS or ZSM-5 has changed in the presence of the other.

### 2.3 Thermogravimetric (TG) analysis

TG measurements were carried out for various PS/ZSM-5 hybrids on a Shimadzu DTG-60 analyzer. For each measurement, about 5.0 mg of the sample was fed and then nonisothermally heated up to 800 K with a heating rate of 5, 10, 15, or 20 K/min. The purging gas was inert nitrogen at a flowing rate of 30 mL/min. The resultant TG data were automatically acquired and its DTG data were readily achieved with the aid of the analysis software.

The pyrolysis performance features may be quantitatively characterized with two pyrolysis parameters, namely, heating resistance index (*HRI*) [33] and comprehensive pyrolysis index (*CPI*) [34], which may be mathematically expressed as the following:


(1) $$HRI=0.49\times [T_{5}+0.6(T_{30}-T_{5})]$$

(2) $$CPI=\frac{{DTG}_{\text{max}}\cdot {DTG}_{mean}}{T_{i}T_{p}\Delta T}$$

where *T*_5_ and *T*_30_ stand for the temperatures when the mass loss is at 5% and 30%, respectively, and both are extracted from the TGA data. *DTG*_max_ and *DTG*_mean_ are defined as the relative maximum mass loss rate (min^−1^) and the average over the entire temperature range (min^−1^) and obtained from the DTG data. T_p_ and T_i_ are the temperature at the maximum mass loss rate and the initial degradation temperature, respectively. ΔT is the temperature range corresponding to DTG/DTG_max_ = 0.5.

### 2.4 Kinetic analysis methods

The pyrolysis decomposition rate of a solid material under nonisothermal conditions can be mathematically represented by differential and integral expressions [6]:


(3) $$\frac{d\alpha }{dT}=\frac{A}{\beta }\text{exp}\left(-\frac{E_{k}}{RT}\right)f(\alpha )$$

(4) $$g(\alpha )=\int_{0}^{\alpha }\frac{d\alpha }{f(\alpha )}=\frac{A}{\beta }\int_{0}^{\text{T}}\text{exp}\left(-\frac{E_{k}}{RT}\right)dT=\frac{AE_{k}}{\beta R}\int_{x}^{\infty }\frac{e^{-x}}{x^{2}}dx=\frac{AE_{k}}{\beta R}p(x)$$

where *A*, *β*, *E_k_*, and *R* represent preexponential factor (min^−1^), heating rate (K/min), activation energy (J/mol) and universal gas constant (8.314 J/mol K), respectively. d*α*/d*t* is the rate of mass conversion (*α*) during pyrolysis process and *α* is the extent of solid mass conversion defined as *α* = (*m_i_* − *m_t_*)/(*m_i_* − *m_e_*) and m*_t_*, m_i_ and *m_e_* are respectively the sample mass at time *t*, original mass, and ultimate mass of the sample, and they are directly abstracted from experimental TG data. *f*(*α*) and *g*(*α*) are the reaction mechanism models in its differential and integral form, respectively, usually assumed to be dependent on mass conversion only and some of these models [35] are listed in Table 1. In the meantime, *x* = *E_k_*/*RT* and $p(x)=\int_{x}^{\infty }\frac{e^{-x}}{x^{2}}dx$. Because *p*(*x*) does not have an analytical solution, numerous approximate solutions or numerical regressions have been proposed. Till now, a lot of integral methods have thus been obtained for kinetic calculations and some mathematical equations for FWO [23, 24], CR [25], MKN [26], SK [27] and TLZW [28] methods are listed in Table 2. It may be noticed that the preexponential factor *A* stands for the impact frequency of molecules or vibration frequency of chemical bonds in the thermal degradation process while *E*_k_ represents the minimum energy required for initiating pyrolysis reaction. ICTAC Kinetics Committee [6] has suggested that the pyrolysis process may be considered as a single-step reaction and characterized by a specific reaction model when there is a little variation of *E*_k_ with *α*.

Apart from linear integral methods, Vyazovkin and Dollimore [29] have proposed a nonlinear integral method, i.e. the VD method, to more accurately calculate the isoconversional activation energy. For each *α*, *E_k_* is regressed by minimizing the following objection function *S*:


(11) $$S=\left|n\left(n-1\right)-\sum_{\text{i}\neq \text{j}}^{\text{n}}\sum_{\;}^{\text{n}}\frac{I\left(E_{k},T_{i}\right)\beta_{j}}{I\left(E_{k},T_{j}\right)\beta_{i}}\right|,$$

where *n* is the number of different heating rates used and *I*(*E_k_*,*T*) is defined as $I(E_{k},T)=\int_{0}^{\text{T}}\text{exp}(-\frac{E_{k}}{RT})dT$, and can be numerically regressed or calculated with a very accurate approximation with the aid of MATLAB software. At present work, a fourth order of Senum-Yang approximation [36] is adopted to be *Q*(*x*) as follows:


(12) $$Q\left(x\right)=\frac{\text{exp}(-x)(x^{4}+18x^{3}+86x^{2}+96x)}{x^{2}(x^{4}+20x^{3}+120x^{2}+240x+120)},$$

where *Q*(*x*) = *R*/*E*_k_·*I*(*E_k_*,*T*).

In Table 2, a differential Friedman method is also included and in short it is called FD method. This isoconversional method is directly derived from Eq. 3 without any approximation or assumption, and then becomes the most commonly used method for any thermal decomposition.

For better describing pyrolysis of polystyrene, the most suitable reaction mechanism function *g*(*α*) as a second kinetic parameter must be determined, and in present work the master plots method suggested by Gotor et al. [31] has been attempted as below:


(13) $$\frac{g(\alpha )}{g(0.5)}=\frac{p(x)}{p(x_{0.5})},$$

where *x_0.5_* is denoted to be *E_k_*/*RT_0.5_*, and *T*_0.5_ is pyrolysis temperature at *α* = 0.5. Based on Eq. 4, the authors have introduced a reference point of *g*(0.5) at *x_0.5_* that can be easily derived as the following:


(14) $$g(0.5)=\frac{AE_{k}}{\beta R}p(x_{0.5}),$$

Clearly, on the left side of Eq. 13, the term *g*(*α*)/*g*(0.5) is a reduced theoretical term, consequently leading to a *g*(*α*)/*g*(0.5)–*α* curve for any reaction model *g*(*α*). Interestingly, for all theoretical models listed in Table 1, *g*(*α*)/*g*(0.5) at *α* = 0.5 should be equal to 1. In other words, all *g*(*α*)/*g*(0.5)–*α* theoretical curves will pass through the point of (0.5, 1.0). On the other hand, on the right of Eq.13,*p*(*x*)/*p*(*x*_0.5_) is an experimental term. Using the *E*_k_ values calculated via the VD method along with the data of (*α*, *T*), *p*(*x*)/*p*(*x*_0.5_) can be obtained for any *α*. By carefully comparing different theoretical master plots of *g*(*α*)/*g*(0.5)–*α* against the experimental master plots of *p*(*x*)/*p*(*x*_0.5_)–*α*, the most suitable reaction model can be resulted if theoretical values are equal to experimental ones. There are two points worth noting here about the master plots method. One is that the above comparison should be made for each heating rate since different heating rate may result in different *p*(*x*)/*p*(*x*_0.5_) values otherwise the experimental values may be averaged before making such comparison. The other one is that when deriving Eq. 13 from Eqs. 4 and 14, *A* and *E*_k_ are explicitly taken as a constant in the whole conversion range even though such assumptions are often unsatisfied.

Considering such an embarrassing case, a compensation effect aided TZLW procedure has been here attempted to further validate the reaction model suggested by the master plots method, apart from accurately yielding the ln*A*. The compensation effect means that there is a linear relationship between ln*A* and *E*_k_, as written below:


(15) $$\text{ln}A=aE_{k}+b,$$

where *a* and *b* are two characteristic compensation parameters. Once both *a* and *b* are available, accurate ln*A* can be evaluated by substituting accurate *E*_k_ values. Furthermore, the respective pairs of ln*A* and *E*_k_ from any method can be fitted into Eq.15, leading to two parameters of *a* and *b*. More recently, Vyazovkin has highlighted that using all ln*A* and *E*_k_ pairs, correct or not, such approach is highly accurate to yield *a* and *b* for both kinetic single-stage and multistage pyrolysis processes [32].

Following this procedure, denoted here as the VT/CE method, the single heating-rate data are considered via Eq. 9 in Table 2. At first, using the *E*_k_ values from the VD method and a given *g*(*α*) suggested by master plots method, the *α*-dependent lnA values can be estimated and four groups of these values are obtained for four heating rates but the differences among these groups are limited. According to the compensation effect, the ln*A* and *E*_k_ data over four heating rates are drawn together for each *g*(*α*). The data will be condensed on a single line once the most suitable reaction model is used. It may be noticed that the closer to 1.0 the linearity coefficient (*R*^2^) for the resultant line, the more suitable the corresponding reaction model for describing pyrolysis of PS samples. Once a *g*(*α*) function is determined to be the most probable, the ln*A* has been obtained simultaneously as well.

## 3. Results and discussion

### 3.1 XRD result analysis

**Figure 1:**
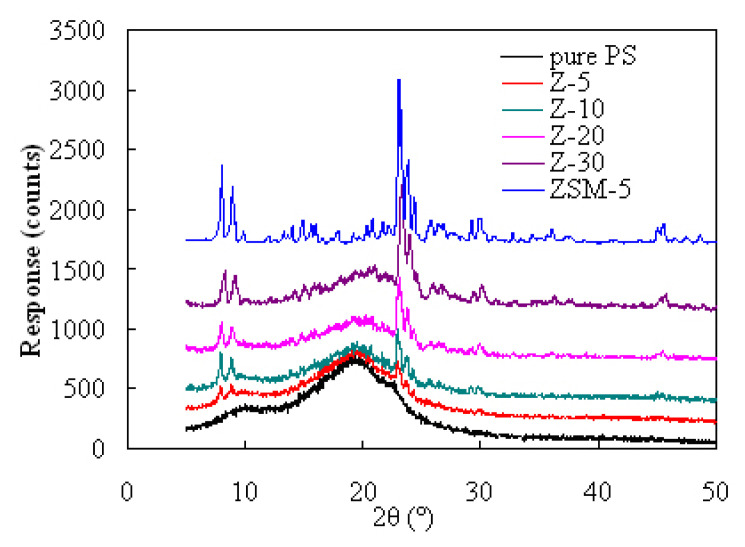
XRD spectra acquired for zeolite ZSM-5, pure PS and their PS/ZSM-5 hybrid samples.

Figure 1 shows XRD results determined for pure PS, zeolite ZSM-5 powder and their three PS/ZSM-5 hybrids at a zeolite loading of 5, 10, 20, and 30 wt.%. As reflected by the XRD patterns, zeolite ZSM-5 shows its strongest peaks with 2*θ* around 7.4–9.4° and 22.8–24.6°, consistent very well with typical MFI topological features [21]. In contrast, pristine PS tends to exhibit not any crystalline peak but a broad hump from 2*θ* = 13° to 27° in the XRD pattern, demonstrating the amorphous nature of the polymer microstructure. The characteristic peaks of zeolite ZSM-5 are clearly seen to keep very well, and the intensity of these peaks becomes stronger as the zeolite content augments in the PS hybrids, as reflected by the XRD patterns. Hence, it may be deduced that there is no crystalline structure variation for zeolite entitles after embedded in the polymer matrix. Similarly, the amorphous hump XRD patterns of PS are also observed as well, confirming that the polymer microstructure may have not been affected by the presence of the added zeolite.

### 3.2 Pyrolysis analysis of PS/ZSM-5 hybrids

**Figure 2:**
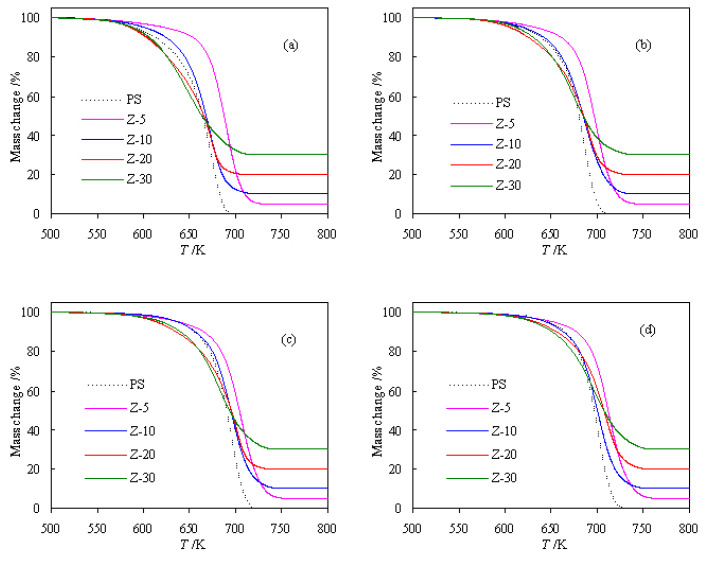
Pyrolysis TGA results of four PS/ZSM-5 hybrids: a) 5 K/min, b) 10 K/min, c) 15 K/min, and d) 20 K/min.

Figure 2 shows the normalized thermogravimetric curves of three PS/ZSM-5 hybrids obtained under four heating rates of 5, 10, 15, and 20 K/min. It can be seen that for Z-10, Z-20, and Z-30 samples, the total mass loss at the end of pyrolysis is almost close to 90, 80, and 70 wt.%, respectively. The result is consistent with the amount of zeolite ZSM-5 used to prepare three samples, i.e. 10, 20, and 30 wt.%, respectively. The reason for it is that the zeolite used will not undergo thermal decomposition under experimental heating conditions, and hence it will retain as the final solid residue at the end of PS pyrolysis. Certainly, such observation is a natural occurrence of inorganic species under conventional heating conditions [16]. Figure 2 clearly shows that the mass loss of four PS/ZSM-5 hybrids mainly occurs in the range of 621.2–740.0 K, 598.1–728.3 K, 581.2–730.7 K, and 582.1–735.8 K, respectively.

For the convenience of comparison, the pyrolysis mass-loss curve of pure PS [14] is also plotted together with the pyrolysis curves of PS-ZSM-5 hybrids. From Figure 2, it can be clearly observed that the thermal stability of PS has been improved to a certain extent after adding 10 wt.% ZSM-5, and the pyrolysis temperature has moved slightly to the high temperature zone as compared to the pure PS case. On the other hand, when 20 wt.% ZSM-5 is added, the thermal stability of PS becomes deteriorated to a certain extent, and the pyrolysis temperature has shifted slightly to the low temperature range. Furthermore, after adding 30 wt.% ZSM-5, the pyrolyzing curve of PS has considerably shifted to the right and it becomes more significantly instable than pure PS under the same pyrolysis condition. These results tend to suggest that higher zeolite loading tends to have a catalytic effect on PS pyrolysis and lower ZSM-5 zeolite loading could improve the thermal stability of PS. For better elucidating such finding, a PS hybrid with 5 wt.% ZSM-5 content, namely as Z-5 sample, is also considered and its pyrolysis analysis has been investigated as well. The resultant TGA results are also shown in Figure 2. As a result, the thermal stability of PS has been enhanced more significantly by adding 5 wt.% ZSM-5 than 10 wt.% ZSM-5. Very similarly, Laachachi et al. [37] have investigated the effect of TiO_2_ on thermooxidative degradation of poly(methyl methacrylate) (PMMA) and found that the addition of 5 wt.% of TiO_2_ into PMMA can stabilize it by more than 40 K but for higher loadings, a catalytic effect on PMMA degradation is observed and become stronger at higher TiO_2_ content. Likewise, Japić et al. [38] have reported that the ZnO content of 0.05–0.15 wt.% has stabilized PMMA by shifting the degradation interval toward higher temperatures and increasing the apparent activation energy relative to pure PMMA whereas at higher content, the catalytic effect of ZnO on PMMA degradation starts to prevail as reflected by lower degradation temperature ranges and lower apparent activation energy.

**Figure 3:**
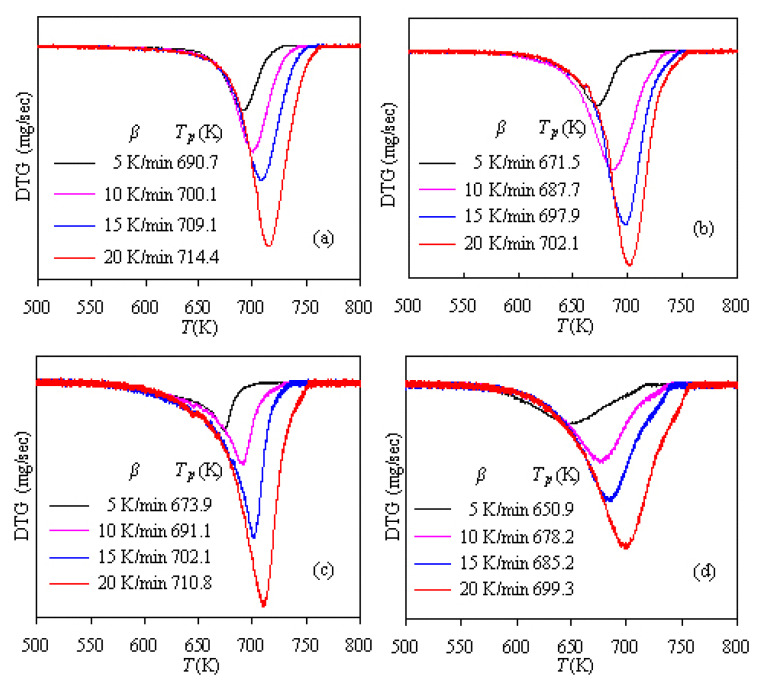
DTG curves of four PS/ZSM-5 hybrids under different heating rates: a) Z-5, b) Z-10, c) Z-20, and d) Z-30.

Figure 3 presents the DTG curves and trough temperatures of *T*_p_ for four PS-ZSM-5 hybrids. It can be seen that, for each sample, there is only one peak in the DTG curves and the *T*_p_ value rises up as *β* increases. For example, the *T*_p_ values for Z-10 are 671.5, 687.7, 697.9, and 702.1 K at *β* = 5, 10, 15, and 20 K/min, respectively. In the case of the same heating rate, the *T*_p_ values of four samples are dropping in the sequence of Z-30 < Z-10 < Z-20 < Z-5, and taking 5 K/min as an example, the *T*_p_ values are 650.9, 671.5, 673.9, and 690.7 K for four PS hybrids, respectively.

Table 3 presents the pyrolysis performance parameters calculated for four PS/ZSM-5 zeolite samples, and it is seen that *T*_5_, *T*_30_ and *T*_i_ for each hybrid go up with the increase in heating rate, similar to the dependence of *T*_p_ on the heating rate as discussed above. It is understandable since during pyrolysis process heat and mass transfers are heavily affected as the heating rate increases, and some components inside the sample may not gasify promptly, subsequently leading to conspicuous thermal hysteresis. Such observation seems to agree very well with the increased thermal resistance, i.e. *HRI*, with the elevated heating rate [33]. Additionally, the *HRI* value is found to increase in the sequence of Z-30 < Z-20 < Z-10 < Z-5, indicating that the effects of different zeolite contents on the pyrolysis feature of PS are substantially different. Table 3 also shows that both *DTG*_max_ and *DTG*_mean_ become enhanced at the elevated heating rate, seemingly suggesting that more volatiles were discharged from the sample and the pyrolysis became easy as the heating rate was raised. This finding may be elaborated by using *CPI* to evaluate the material pyrolyzability. The larger the *CPI* value, the easier the pyrolysis and the better the pyrolysis performance [34]. As shown in Table 3, the *CPI* values are augmented considerably as the heating rate elevates, further suggesting the more favorable pyrolysis degradation.

### 3.3 Kinetic analysis of pyrolysis of PS/ZSM-5 hybrids

#### 3.3.1 Calculation of *E*_k_

**Figure 4:**
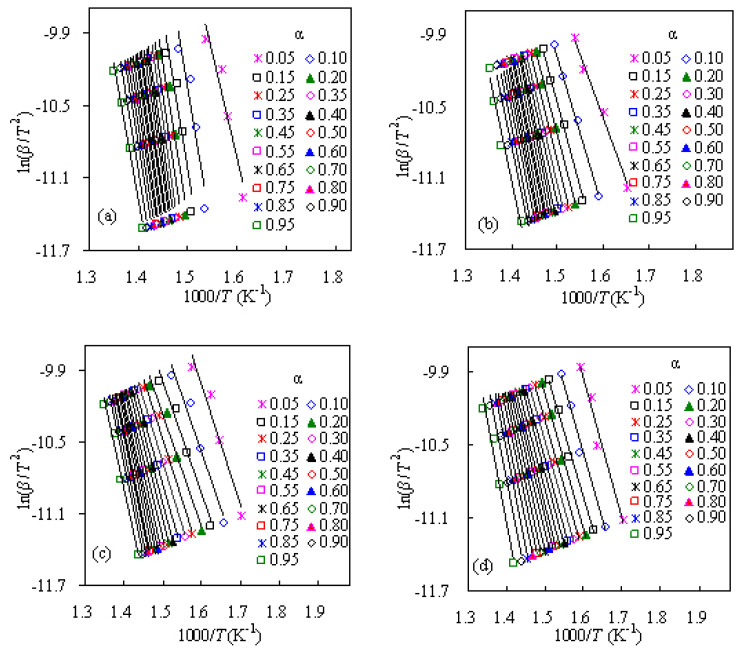
The CR plots of ln(*β*/*T*^2^)–1000/*T* for pyrolysis of four PS/ZSM-5 hybrids: a) Z-5, b) Z-10, c) Z-20, and d) Z-30.

**Figure 5:**
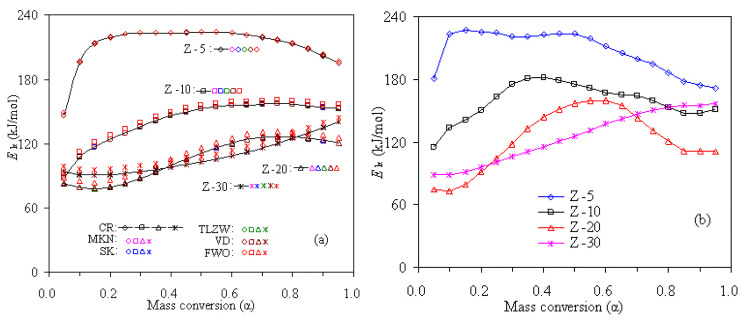
Mass conversion dependent *E*_k_ calculated for four PS/ZSM-5 hybrids: a) integral methods, and b) differential FD method.

The activation energies of PS-ZSM-5 hybrids are calculated with the use of CR method and the Arrhenius plots of ln(*β*/*T*^2^)–1000/*T* are presented in Figure 4. From the slopes of these linear plots, all the mass conversion-dependent activation energies can be obtained and the calculated *E*_k_ values are presented in Figure 5a for four PS/ZSM-5 hybrid samples. Similarly, linear FWO, MKN, SK, and TLZW methods are also attempted to calculate the activation energies and the results are also graphically presented in Figure 5a.

A careful examination indicates that these integral methods, including the VD method discussed in detail later, generally generate rather close values for all the *E*_k_ of the same mass conversional level. For instance, the averaged *E*_k_ resulted from FWO, CR, MKN, SK, TLZW and VD methods are 145.3, 141.5, 141.9, 141.9, 141.9, and 141.9 kJ/mol for Z-10, respectively. As addressed earlier, these methods involve different approximation treatments to the temperature integral function, but here they are equal in accuracy to result in *E*_k_ values. Overall, the deviations in *E*_k_ among the CR, MKN, SK, TLZW, and VD methods are not higher than 0.5 kJ/mol for the same α value. On the contrary, the *E*_k_ values from the FWO method are seen to deviate by 2.5–5.0 kJ/mol as compared to those from the other methods. By comparing *E*_k_ values of four PS hybrids, it can be seen from Figure 5a that the *E*_k_ of Z-30 increases gradually with mass conversion while the *E*_k_ of the other hybrids appears to climb up to a flat plateau and then decrease as α boosts up continuously. Such difference suggests that their pyrolysis processes may have followed different degradation mechanisms, which will be discussed later.

In addition to the linear methods mentioned above, the nonlinear temperature integral VD method is also attempted to simulate the *E*_k_ for pyrolysis of PS/ZSM-5 hybrids and the resultant *E*_k_ values are given in Figure 5a as well. As can be seen from the table, the *E*_k_ ranges from 146.2 to 223.9 kJ/mol for Z-5, 87.4 to 141.9 kJ/mol for Z-10, 83.2 to 106.1 kJ/mol for Z-20, and 94.1 to 107.7 kJ/mol for Z-30, respectively. If averaged over the entire conversion range, the *E*_k_ values are 212.5, 141.9, 122.6 and 124.7 kJ/mol for Z-5, Z-10, Z-20 and Z-30, respectively. Our previous study [14] shows that the averaged *E*_k_, obtained by VD method, is 139.5 kJ/mol for pure PS. Clearly, the averaged *E*_k_ values of Z-5 and Z-10 are higher than that of pure PS while the other two PS/ZSM-5 hybrids of high zeolite loading have lower *E*_k_ than pure PS, seemingly suggesting that the zeolite ZSM-5 content has surprising effect on the *E*_k_ of PS pyrolysis. Further comparison shows that the *E*_k_ values have varied in the order of Z-5 > Z-10 > Z-30 ≈ Z-20. If compared with the *E*_k_ of pure PS, it can be found that for every *α*, Z-5 has larger *E*_k_ than pure PS, indicating that the addition of 5 wt.% ZSM-5 plays a stabilizing role on PS, making the PS pyrolysis more difficult. For the case of Z-10 hybrid, it has higher *E*_k_ than pure PS as *α* < 0.5 and the opposite is obtained when *α* > 0.5. These results suggest that the addition of 10 wt.% ZSM-5 has also made PS more heat-resistant, but the stabilization effect become lower than the case of adding 5 wt.% ZSM-5. As for Z-20, it exhibits lower *E*_k_ than pure PS when *α* > 0.10 and for Z-5 its *E*_k_ is lower than that of pure PS when *α* > 0.15. These results indicate that the addition of 20 or 30 wt.% ZSM-5 has reduced the activation energy of PS pyrolysis and turned to play a considerable catalytic effect, consistent very well with the pyrolysis temperature discussed previously. Likewise, similar opposite effects of critically adding ZnO on thermal degradation of PMMA have also been reported by Japić et al. [38], where the authors have stated that catalytic effect can prevail over the stabilizing effect due to increased ZnO/PMMA interface at higher ZnO loading. In the meantime, Laachachi et al. [37] have observed that there is an increase in *E*_k_ for 5 wt.% TiO_2_ added PMMA compared to pure PMMA corresponding to the stabilizing effect and there is a decrease in *E*_k_ when the TiO_2_ loading increases corresponding to the catalytic effect. Besides, they also observe that nanosized TiO_2_ has stronger catalytic effect than micrometric TiO_2_ due to the increase in the surface contact between particle and matrix. Therefore, it may be thought that the porosity of zeolite entities may have contributed very much to the effect on PS pyrolysis observed in present work. Here, ZSM-5 zeolite may act as a “physical” cross-linking agent in the PS hybrids due to the partial invasions of polymer chains into zeolitic framework or the partial pore occlusions, subsequently leading to polymer chain rigidification and partial pore blockage [39,40]. Then, the presence of ZSM-5 zeolite may have rendered the PS hybrids become thermally rigid and take a stabilizing effect on PS pyrolysis, and such thought is possibly true for the Z-5 sample. However, at higher ZSM-5 zeolite loading of 20–30 wt.%, the PS/ZSM-5 interfacial area has substantially increased, and the enhanced interfacial contact has then facilitated thermal decomposition of PS [37]. Such facilitation impact may dominate over the physical cross-linking effect for the higher ZSM-5 zeolite loading, subsequently leading to the transition of stabilizing effect at 5 wt.% ZSM-5 loading to catalytic impact at 20–30 wt.% ZSM-5 loading.

**Figure 6:**
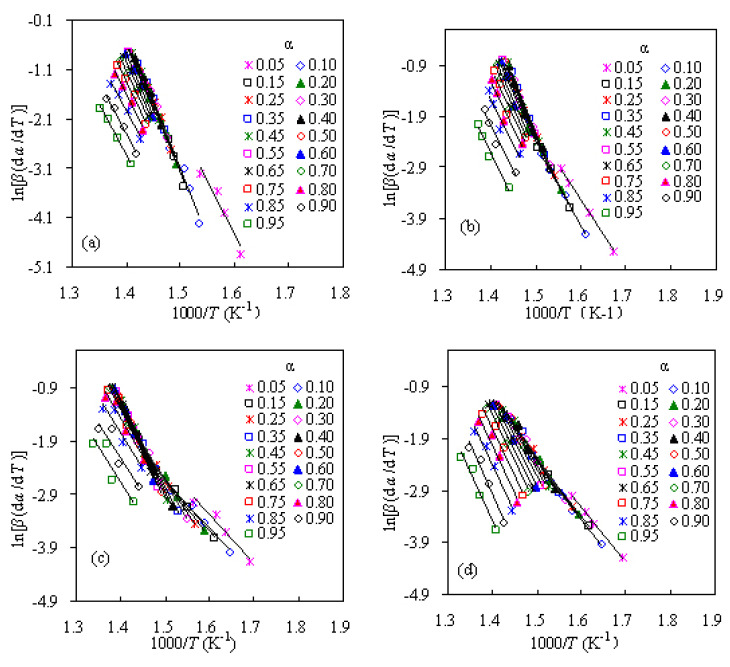
The FD plots of ln[*β*/(d*α*/d*T*)]–1000/*T* for pyrolysis of four PS/ZSM-5 hybrids: a) Z-5, b) Z-10, c) Z-20, and d) Z-30.

The *E*_k_ results calculated by the linear differential FD method are shown in Figure 5b and the Arrhenius plots of ln(*β*(d*α*/d*T*)–1000/*T* involved are presented in Figure 6. Apparently, the *E*_k_ results by the FD method are distinct from those obtained from the other integral methods, because 1) the FD method is the exact differential form while the others are the integral expressions with certain extent of approximation, and 2) the *E*_k_ values from the FD method are inherently dependent on the accuracy of the experimental d*α*/d*T* values.

#### 3.3.2 Determination of *g*(*α*) and ln*A*

**Figure 7:**
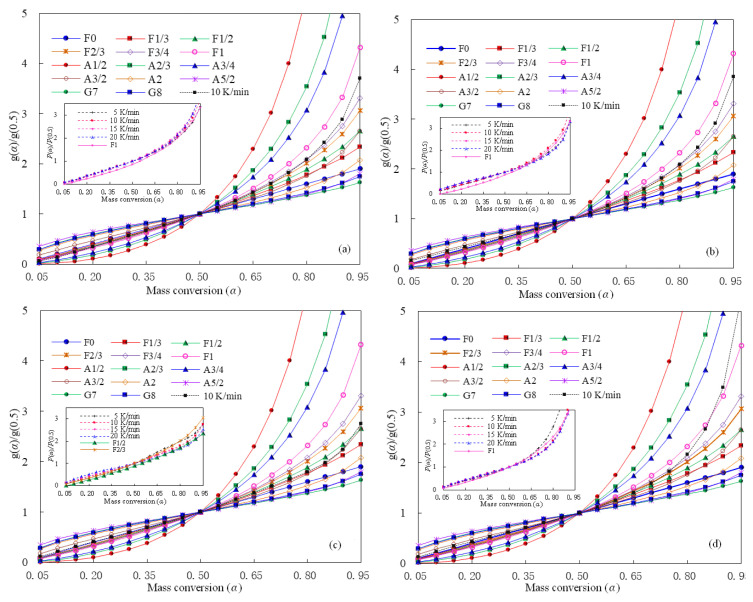
Theoretical and experimental Master plots obtained at 10 K/min for four PS/ZSM-5 hybrid samples: a) Z-5, b) Z-10, c) Z-20, and d) Z-30.

In present study, the master plots method has been attempted for preliminarily scanning the well-performed reaction model for describing pyrolysis of polystyrene hybrids. It may be noted that the single-step reaction model is globally considered as a straightforward method for describing pyrolysis process and has been done here for PS hybrids because that 1) there is one DTG peak resulted from each heating rate for all hybrid samples; 2) the deviation of α-dependent *E*_k_ from the averaged *E*_k_ is not very large and the averaged deviation of 5.6, 11.0, 16.2, and 12.4% are respectively for Z-5, Z-10, Z-20, and Z-30 samples; and 3) a better comparison can be made between pure PS and its ZSM-5 hybrids when using the one-step reaction model assumption as that done for pure PS [14]. Using the *E*_k_ obtained from the VD method, the model-fitting master plots can be resulted and presented in Figure 7 for four PS/ZSM-5 hybrids. The *g*(*α*)/*g*(0.5)–*α* curves involved in the master plots are theoretical curves resulting from the pyrolysis mechanism functions given in Table 1. In contrast, the experimental *p*(*x*)/*p*(*x*_0.5_) curve is somewhat influenced by the heating rate *β* according to Eq. 13, and the embedded images in Figure 7 depict the experimental *p*(*x*)/*p*(*x*_0.5_)–*α* curves for different *β* cases. As observed, the *p*(*x*)/*p*(*x*_0.5_) values are different more or less under the heating rates of 5, 10, 15, or 20 K/min but they are basically the same. For simplicity, the experimental *p*(*x*)/*p*(*x*_0.5_) curve correspondent to 10 K/min is taken as an example in the master plots for PS/ZSM-5 hybrids. As observed in Figure 7, none of the theoretical master plots could completely be condensed on the experimental curves for any PS/ZSM-5 hybrid. However, one may see that the experimental curves of Z-5, Z-10 and Z-30 are very close to the theoretical master plots of F1. For Z-20, its experimental curve is close to the theoretical F2/3 curve when α < 0.5, and in-between the theoretical F1/2 and F2/3 curves when α > 0.5. These results indicate that pyrolysis of PS/ZSM-5 hybrids may follow the chemical reaction mechanism of different reaction orders. In addition, it can be found that the *p*(*x*)/*p*(*x*_0.5_)–α curves of PS-ZSM-5 hybrids and some Avrami-Erofeev reaction function curves are relatively close to each other. Therefore, further analysis is performed so as to find the most appropriate reaction function from the above two types of mechanisms to describe pyrolysis behaviors of PS/ZSM-5 hybrids.

**Figure 8:**
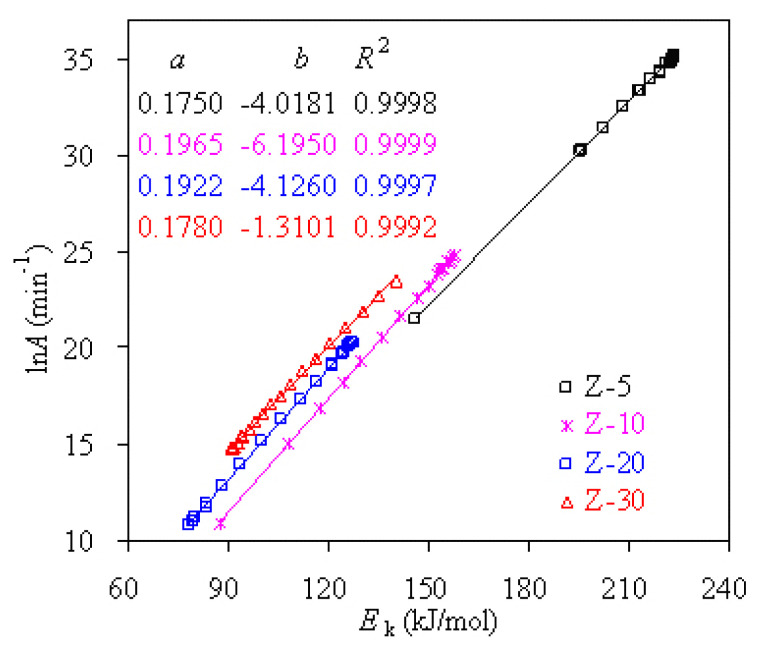
The compensation relationship between ln*A* and *E_k_* for pyrolysis of four PS/ZSM-5 hybrids over the entire conversion range.

Based on the findings scanned with the master plots method, further validation has been performed by using the VT/CE method and the final results are graphically given in Figure 8 for four PS-ZSM-5 hybrids. The resultant *R*^2^ values from the most probable mechanism models are clearly shown in Figure 8 and these models with *R*^2^ very close to 1.0 are F0.92, F0.85, F0.56 and A1.32 for Z-5, Z-10, Z-20, and Z-30, respectively. It may be deduced that they are the most appropriate pyrolysis mechanism functions for four PS-ZSM-5 hybrids.

With the VT/CE method, ln*A* can be accurately estimated according to the compensation effect [32, 41]. Using two compensation effect parameters of *a* and *b* also given in Figure 8, the ln*A* values are calculated by substituting *E*_k_ into Eq. 15. The averaged ln*A* for Z-5 is 35.62 min^−1^, correspondent to an *A* number of 1.05 × 10^16^ min^−1^. Likewise, the averaged ln*A* values are 23.51, 16.91 and 17.62 min^−1^ for Z-10, Z-20, and Z-30 hybrids, respectively. Correspondingly, their *A* values are then 1.44 × 10^11^, 3.74 × 10^8^, and 1.88 × 10^9^ min^−1^, respectively. Looking at these numbers, the order of the *A* values is the same as that of *E*_k_ and *A* is decreasing in sequence of Z-5 > Z-10 > Z-30 > Z-20.

#### 3.3.3 Reestablishing *α–T* curves for pyrolysis of PS/ZSM-5 hybrids

**Figure 9:**
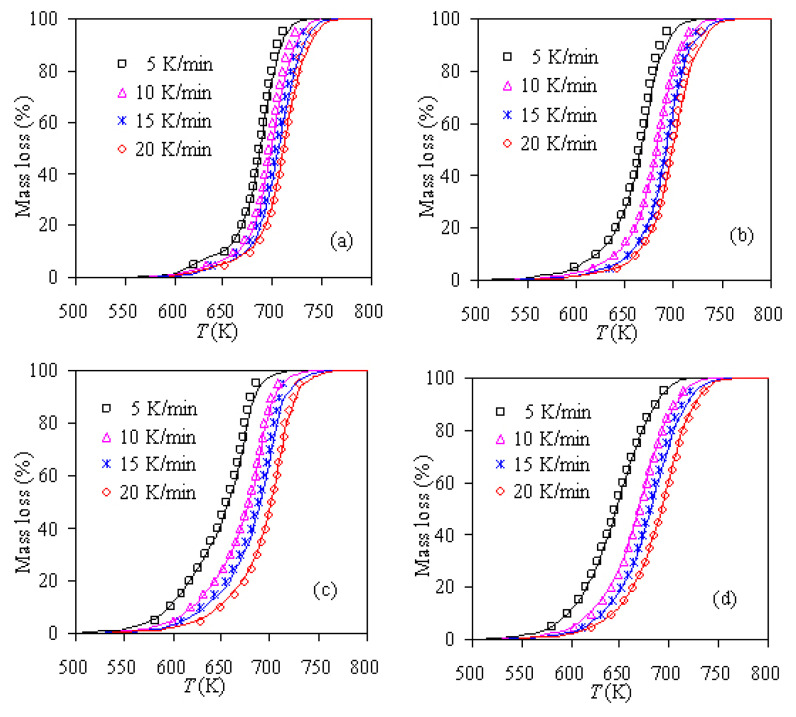
Reestablished pyrolysis *α–T* curves for four PS/ZSM-5 hybrid samples: a) Z-5, b) Z-10, c) Z-20, and d) Z-30.

With the kinetic tri-parameters of *E*_k_, *A* and *f*(*α*), the *α–T* curves and differential d*α*/d*T–T* curves may be rebuilt, and the resultant curves of *α–T* are shown in Figure 9 for four PS/ZSM-5 hybrids. The performances of the simulations are analyzed by comparing with the experimental data, and thus, the experimental results for four hybrids are also presented in Figure 9 for better comparison. It can be clearly seen that most of the calculated results are basically coinciding with the experimental data for pyrolysis of PS/ZSM-5 hybrids, and such consistence indicates that the theoretical model can well reflect the temperature-dependence of the mass conversion. Such satisfactory results are exciting for our theoretical calculations even though at high conversion levels, there are some deviations between the calculated and experimental values, especially for Z-5 and Z-10 hybrids. In addition, the differential d*α*/d*T–T* curves can be recast by using the functions of d*α/*d*t* = 1.05 × 10^16^·exp( − 2.556 *×* 10^4^/*T*)·(1 − *α*)^0.92^, 1.44 × 10^11^·exp( − 1.707 *×* 10^4^/*T*)·(1 − *α*)^0.85^, 3.74 × 10^8^·exp(−1.475 *×* 10^4^/*T*)·(1 − *α*)^0.56^, and 2.48 × 10^9^·exp( − 1.499 *×* 10^4^/*T*)·(1 − *α*)·[ − ln(1 − *α*)]^0.242^ min^−1^ for Z-5, Z-10, Z-20, and Z-30, respectively.

## 4. Conclusion

In this paper, thermogravimetric analysis has been used to measure the pyrolysis behaviors of the hybrids of PS and zeolite ZSM-5, and kinetic analysis is detailed for describing the pyrolysis features. The calculations of three kinetic parameters have been attempted by using a variety of isoconversional methods. Some conclusions of present work may be given as follows:

1. TGA results show that the addition of 20 and 30 wt.% zeolite ZSM-5 has significantly reduced the initial pyrolysis temperature of PS, indicating that zeolite plays a significant catalytic effect. On the other hand, the addition of 5 and 10 wt.% zeolite ZSM-5 has promoted the initial pyrolysis temperature of PS, indicative of the stabilization effect of ZSM-5 on the PS pyrolysis stability. With the increase of zeolite ZSM-5 content, the effect of ZSM-5 on the PS pyrolysis has transferred from stabilizing to catalyzing, and such finding has not been reported and the reason for it is unclear yet.
2. The nonisothermal TGA data obtained under 5, 10, 15, and 20 K/min are kinetically analyzed for PS/ZSM-5 hybrids with a number of isoconversional methods. The results show that the activation energies obtained by integral VD, CR, MKN, SK and TLZW methods are very close to each other provided that the mass conversion *α* is the same, while the values from the integral FWO method are relatively larger although the *E*_k_–*α* dependence trend is exactly the same for six integral methods. However, the *E*_k_ values and the dependence of *E*_k_ on *α* by the differential FD method are obviously different.
3. The addition of 5 or 10 wt.% zeolite ZSM-5 has increased the pyrolysis *E*_k_ and enhanced the thermal stability of PS, while the average *E*_k_ has reduced after the addition of 20 and 30 wt.% ZSM-5 into PS, and high zeolite ZSM-5 content has catalytically made the PS pyrolysis undergo more easily.
4. The integrated master plots and VT/CE method has been proposed and successfully attempted to achieve the most probable *f*(*α*) for pyrolysis of PS/ZSM-5 hybrids. The results show that F0.92 and F0.85 are the most appropriate model to describe the pyrolysis of Z-5 and Z-10 while F0.56 and A1.32 are for Z-20 and Z-30, respectively. With three kinetic parameters of *E*_k_, ln*A* and *f*(*α*), excellent performances have been achieved as reflected by multiple *α–T* curves successfully reestablished over the entire conversion range for four PS/ZSM-5 hybrids.

## Figures and Tables

**Table 1:** Selected reaction models used for describing pyrolysis of PS/ZSM-5 hybrids.

Model	*g*(*α*)	*f*(*α*)	Rate-determining mechanism
F0	*α*	1	Chemical reaction
F1/3	1 − (1 − *α*)^2/3^	(3/2)(1 − *α*)^1/3^	Chemical reaction
F1/2	1 − (1 − *α*)^1/2^	2(1 − *α*)^1/2^	Chemical reaction
F2/3	1 − (1 − *α*)^1/3^	3(1 − *α*)^2/3^	Chemical reaction
F3/4	1 − (1 − *α*)^1/4^	4 (1 − *α*)^3/4^	Chemical reaction
F1	−ln(1 − *α*)	1 − *α*	Chemical reaction
Fn (n≠1)	[(1 − *α*)^1 − n^ −1]/(n − 1)	(1 − *α*)^n^	Chemical reaction
A1/2	[− ln(1 − *α*) ]^2^	(1/2)(1 − *α*)[ − ln(1 − *α*)] ^−^^1^	Random nucleation
A2/3	[− ln(1 − *α*) ]^3/2^	(2/3)(1 − *α*)[ − ln(1 − *α*)] ^−^^1/2^	Random nucleation
2A3/4	[− ln(1 − *α*) ]^4/3^	(3/4)(1 − *α*)[ − ln(1 − *α*)] ^−^^1/3^	Random nucleation
A3/2	[− ln(1 − *α*) ]^2/3^	(3/2)(1 − *α*)[ − ln(1 − *α*)]^1/3^	Random nucleation
A2	[− ln(1 − *α*) ]^1/2^	2(1 − *α*)[ − ln(1 − *α*)]^1/2^	Random nucleation
A5/2	[− ln(1 − *α*) ]^2/5^	(5/2)(1 − *α*)[ − ln(1 − *α*)]^3/5^	Random nucleation
An (n≠1)	[− ln(1 − *α*) ]^1/n^	n(1 − *α*)[ − ln(1 − *α*)]^(1^^−^^1/n)^	Random nucleation
G7	[1 − (1 − *α*)^1/2^]^1/2^	4(1 − *α*)^1/2^[1 − (1 − *α*)^1/2^] ^1/2^	Kinetic equations with unjustified mechanisms
G8	[1 − (1 − *α*)^1/3^]^1/2^	6(1 − *α*)^2/3^[1 − (1 − *α*)^1/3^]^1/2^	

**Table 2:** Some isoconversional methods considered in present work.

Method	Mathematical expression	Eq.	Ref.
FWO	$\text{log}\beta =\text{log}\left[\frac{AE_{k}}{Rg(\alpha )}\right]-0.4567\frac{E_{k}}{RT}-2.315$	(5)	[23, 24]
CR	$\text{ln}\left(\frac{\beta }{T^{2}}\right)=\text{ln}\left[\frac{AR}{E_{k}g(\alpha )}\right]-\frac{E_{k}}{RT}$	(6)	[25]
MKN	$\text{ln}\left(\frac{\beta }{T^{1.884318}}\right)=\text{ln}\left[\frac{A}{g(\alpha )}{\left(\frac{E_{k}}{R}\right)}^{-0.884318}\right]-1.001928\frac{E_{k}}{RT}-0.389677$	(7)	[26]
SK	$\text{ln}\left(\frac{\beta }{T^{1.92}}\right)=\text{ln}\left[\frac{A}{g(\alpha )}{\left(\frac{E_{k}}{R}\right)}^{-0.92}\right]-1.0008\frac{E_{k}}{RT}-0.312$	(8)	[27]
TLZW	$\text{ln}\left(\frac{\beta }{T^{1.894661}}\right)=\text{ln}\left[\frac{A}{g(\alpha )}{\left(\frac{E_{k}}{R}\right)}^{-0.894661}\right]-1.00145033\frac{E_{k}}{RT}-0.37773896$	(9)	[28]
FD	$\text{ln}\left[\beta \left(\frac{d\alpha }{dT}\right)\right]=\text{ln}\left[Af(\alpha )\right]-\frac{E_{k}}{RT}$	(10)	[30]

**Table 3:** Pyrolysis performance parameters for PS/ZSM-5 hybrids under different heating rates.

Sample	*β*(K/min)	*T*_5_ (K)	*T*_30_ (K)	*T*_i_ (K)	*T*_P_ (K)	*DTG*_max_ (min^−1^)	*ΔT*(K)	*DTG*_mean_ (min^−1^)	*HRI* (K)	*CPI* (min^−2^K^−3^)
Z-5	5	621.2	677.5	500.0	690.7	−2.614	31.7	−0.2029	320.9	4.84 × 10^−^^8^
	10	632.3	677.5	506.9	700.1	−4.853	31.7	−0.4081	325.6	1.68 × 10^−^^7^
	15	637.6	686.0	502.2	709.1	−5.911	33.3	−0.5456	328.5	2.47 × 10^−^^7^
	20	651.3	692.3	506.8	714.4	−7.985	36.6	−0.7358	333.7	4.42 × 10^−^^7^
Z-10	5	598.1	653.1	517.9	671.5	−0.113	34.2	−0.0093	309.3	8.82 × 10^−^^11^
	10	617.2	669.2	510.7	687.7	−0.189	43.7	−0.0187	317.7	2.30 × 10^−10^
	15	634.9	681.7	508.4	697.9	−0.332	35.2	−0.0280	324.9	7.46 × 10^−10^
	20	642.1	687.5	515.4	702.1	−0.447	34.9	−0.0376	328.0	1.33 × 10^−^^9^
Z-20	5	581.2	633.8	501.4	673.9	−0.113	25.5	−0.0102	300.3	1.34 × 10^−10^
	10	600.2	658.0	516.3	691.1	−0.214	30.8	−0.0205	311.1	4.00 × 10^−10^
	15	608.4	669.4	514.9	702.1	−0.349	27.9	−0.0310	316.0	1.07 × 10^−^^9^
	20	627.7	686.0	507.0	710.8	−0.426	34.9	−0.0415	324.7	1.41 × 10^−^^9^
Z-30	5	582.1	628.7	503.0	650.9	−0.073	70.8	−0.0109	298.9	3.44 × 10^−^^11^
	10	603.9	653.0	505.2	678.2	−0.193	53.9	−0.0235	310.4	2.46 × 10^−10^
	15	609.8	663.2	503.2	685.2	−0.254	50.2	−0.0300	314.5	4.40 × 10^−10^
	20	621.0	674.8	508.6	699.3	−0.300	55.8	−0.0377	320.1	5.69 × 10^−10^
